# Global Dynamic Transcriptome Programming of Rapeseed (*Brassica napus* L.) Anther at Different Development Stages

**DOI:** 10.1371/journal.pone.0154039

**Published:** 2016-05-03

**Authors:** Zhanjie Li, Peipei Zhang, Jinyang Lv, Yufeng Cheng, Jianmin Cui, Huixian Zhao, Shengwu Hu

**Affiliations:** 1 State Key Laboratory of Crop Stress Biology in Arid Areas, Northwest A&F University, Yangling, Shaanxi 712100, China; 2 College of Life Sciences, Northwest A&F University, Yangling, Shaanxi 712100, China; 3 College of Agronomy, Northwest A&F University, Yangling, Shaanxi 712100, China; Institute of Botany, Chinese Academy of Sciences, CHINA

## Abstract

Rapeseed (*Brassica napus* L.) is an important oil crop worldwide and exhibits significant heterosis. Effective pollination control systems, which are closely linked to anther development, are a prerequisite for utilizing heterosis. The anther, which is the male organ in flowering plants, undergoes many metabolic processes during development. Although the gene expression patterns underlying pollen development are well studied in model plant *Arabidopsis*, the regulatory networks of genome-wide gene expression during rapeseed anther development is poorly understood, especially regarding metabolic regulations. In this study, we systematically analyzed metabolic processes occurring during anther development in rapeseed using ultrastructural observation and global transcriptome analysis. Anther ultrastructure exhibited that numerous cellular organelles abundant with metabolic materials, such as elaioplast, tapetosomes, plastids (containing starch deposits) etc. appeared, accompanied with anther structural alterations during anther development, suggesting many metabolic processes occurring. Global transcriptome analysis revealed dynamic changes in gene expression during anther development that corresponded to dynamic functional alterations between early and late anther developmental stages. The early stage anthers preferentially expressed genes involved in lipid metabolism that are related to pollen extine formation as well as elaioplast and tapetosome biosynthesis, whereas the late stage anthers expressed genes associated with carbohydrate metabolism to form pollen intine and to accumulate starch in mature pollen grains. Finally, a predictive gene regulatory module responsible for early pollen extine formation was generated. Taken together, this analysis provides a comprehensive understanding of dynamic gene expression programming of metabolic processes in the rapeseed anther, especially with respect to lipid and carbohydrate metabolism during pollen development.

## Introduction

Rapeseed (*Brassica napus* L.) is animportant oil crop worldwide, providing both edible oil and industrial materials such as livestock meal, lubricants and biodiesel [1]. As with many other crops, rapeseed shows significant heterosis, which is the superior performance of hybrids with respect to many agronomic traits relative to their parents. This feature is commonly used to increase crop yields [2], but effective pollination control systems are required for its implementation. In rapeseed, male sterility systems (including cytoplasmic male sterility and genic male sterility), self-incompatibility, and chemical hybridization agents (CHAs) have been experimentally demonstrated to be effective pollination control systems [3], all of which are closely related with the development of the anther. Thus, the study of anther development is foundational for the utilization of heterosis in selective crop breeding.

The anther contains both reproductive cells (pollen mother cells) and non-reproductive cell layers. Its developmental process has been divided into 14 stages according to the morphological features [4] in *Arabidopsis*. From stages 1 to 5, the four lobes of the anther are formed, each consisting of four outer sporophytic cell layers and the innermost gametophytic cells. From the exterior to the interior, the four layers of sporophytic cells are the epidermis, the endothecium, the middle layer, and the tapetum. At stages 6–7, the pollen mother cells (PMCs) undergo meiosis to form tetrads enclosed in a thick shell composed of callose. At stage 8, the callose degrades, and the microspores are released from the tetrads. At stages 9–12, the released microspores go through vacuolization and mitosis to form mature pollen grains. In parallel with anther development and cell division, an abundance of metabolic processes occur. During the early stages of pollen development, developing pollen is immersed in locular fluid containing nutrients, such as sugars and lipids, derived from the sporophytic (somatic) tissue tapetum [5]. During the tetrad stage, the callose, which is composed of β-1,3-glucans, forms and is then degraded in a process involving at least three cell wall enzymes, including β-1,3-glucanase [6,7], endocellulase [8,9], and polygalacturonase (PG) [10]. During pollen maturation, pollen grains accumulate starch as an energy reserve for use during germination, which serves as a marker of pollen maturity [11]. Throughout anther development, pollen wall formation is critical for pollen development, which consists of the following three layers: an inner intine, an outer exine and a lipid- and protein-rich pollen coat in the crevices of the exine. The exine appears at the tetrad stage and is consolidated by tapetally-derived sporopollenin after microspore release from the tetrad, which is a complex polymer primarily composed of fatty acids and phenolic compounds [12]. Meanwhile, the mature stage pollen initiates the formation of intine, which is primarily composed of pectin. Since the anther development process is so complex, numerous genes must be dynamically expressed throughout the process, in large part to coordinate metabolism in both somatic and gametophytic cells [13].

Transcriptomic studies of gene activity at a global level help to elucidate the mechanisms underlying biological processes that occur during anther development. Pollen, which is the haploid male gametophyte of flowering plants, is well studied in terms of gene expression and regulation in *Arabidopsis* and rice. In *Arabidopsis*, Honys and Twell [14], as well as Becker *et al*. [15], firstly investigated the transcriptome of mature pollen using Affymetrix *Arabidopsis* 8K microarrays. They identified 992 [14] and 1587 [15] differentially expressed genes, respectively, in mature pollen, estimating the total number of pollen expressed genes in *Arabidopsis* as being between 3500 and 5500. Honys and Twell [13] further separated the four stages of the microspores (from the uninucleate microspore stage to the mature pollen grain stage) and performed the first dynamic gene expression analysis during male gametophyte development. Their results revealed a phase shift in gene expressions between the bicellular pollen and tricellular pollen stages. In rice, the transcriptome of developing pollen was investigated via laser microdissection of the tapetum, microspores, and pollens using the 44K rice oligo microarray platform [16,17] and the 57K Affymetrix Rice Genome Array [18], respectively. The latter study [18] described a “U-type” change in pollen preferential or stage specific transcripts in rice, with the lowest number of genes preferentially expressed during the bicellular stage. Although large-scale transcriptome profiling studies of pollen have helped to characterize gene expression profiles during pollen development in *Arabidopsis* and rice, it remains unknown how metabolism is regulated to coordinate anther development events occurring both in somatic and gametophytic cells throughout anther development, especially in rapeseed.

In this study, we provide a thorough description of the ultrastructural features and transcriptome during anther development in rapeseed (*B*. *napus*). The ultrastructure exhibited consistent subcellular structures and organelles in anther cells throughout development. A global level transcriptome analysis throughout anther development revealed a dynamic shift in gene expression. Integrative clustering analysis combined with rigorous statistical tests of GO function and predictive gene regulatory networks provided insight into the genes required for cell structure formation and metabolism, especially of lipids and carbohydrates. The anatomical and global RNA profiling analyses mirrored each other, indicating the dynamic regulation of metabolism in the rapeseed anther.

## Materials and Methods

No specific permits were required for the described field studies. No specific permissions were required for these locations/activities. The location is not privately-owned or protected in any way. The field studies did not involve endangered or protected species.

### Plant materials and sample collection

The rapeseed cultivar ‘Zhongshuang No.9’, which was developed by the Oil Crops Research Institute of the Chinese Academy of Agricultural Sciences (Wuhan, China), was planted in an experimental field at Northwest A&F University, Yangling, Shaanxi, China (longitude 108°E, latitude 34°15′N) using optimal agronomic practices.

Samples were collected according to the methods reported by Li *et al*. (2015) [19]. At the time of bolting, the main inflorescences of uniform plants were collected and quickly transported to the laboratory on ice. Acetocarmine staining and light microscopy were performed to examine the correlation of microspore developmental stages with bud length. Then, the buds were classified into three subgroups according to their length: small buds (SBs) with lengths below 1 mm (before and during the pollen mother cell (PMC) stage), medium buds (MBs) 1–3 mm in length (from meiosis to the early uninucleate microspore stage), and large buds (LBs) over 3 mm in length (from the vacuolated microspore to the mature pollen stage). In the MBs and LBs subgroups, anthers were dissected from the buds (referred to as An-MBs and An-LBs, respectively) in order to investigate the transcriptome alterations of rapeseed anther during development. While, in the SBs subgroup, the whole buds were used as samples, because they were too small to dissect anthers. Young leaves (Ls) from the main inflorescences were also collected as a vegetative tissue control for microarray analysis. All samples were prepared on ice, immediately frozen in liquid nitrogen, and then stored at -80°C. Mixed samples collected from approximately 30 different plants were used as one biological replicate, and three independent biological replicates were prepared for each sample.

### Transmission electron microscopy (TEM)

TEM imaging was performed as follows. Anthers at different microspore development stages were treated according to González-Melendi*et al*. [20] for cytological observation. After treatment, the specimens were sectioned using an Ultramicrotome Leica EM UC7 (Leica Microsystems, Germany). Ultrathin sections (70 nm) were observed and photographed with a transmission electron microscope (JEM-1230, JEOl, Tokyo, Japan) on 600 mesh formvar-coated copper grids.

### Microarray hybridization, data acquisition and gene annotation

Global mRNA profiling of leaves and anther samples (SBs, An-MBs and An-LBs) was performed using the Agilent Single Channel Brassica Oligo Microarray (4 × 44 K), which contains 43,803 probe sets designed on the basis of *B*. *napus* ESTs, mRNAs, and predicted gene sequences from databases such as NCBI, TIGRI, and UniGene. The total RNA was isolated and cDNA was synthesized, hybridized and normalized exactly as described previously [19].

The number of mRNAs in each sample was determined using “present (P)” signals. An mRNA was considered present only if it was called “P” in all three biological replicates for each sample. To facilitate global comparisons of gene activity and quantitative analyses of gene expression, the raw data were normalized by a Quantile algorithm [21] using Gene Spring Software 11.0 (Agilent technologies, Santa Clara, CA, US) and were log2 transformed. Finally, the correlations between biological replicates and between different tissues were calculated.

To identify global transcriptome dynamic changes during anther development in *B*. *napus*, Student’s t-test was performed between leaves and anthers at different stages or between anthers at different development stages. The differentially expressed transcripts (DETs) were required to meet the following criteria: 1) a statistically significant difference by Student’s t-test (p value≤0.001), and 2) an expression alteration cut-off of 2-fold.

Due to the limited gene annotation information available for *B*. *napus* and the high similarity (approximately 85%) of coding sequence between *B*. *napus* and *A*. *thaliana* [22], we annotated the identified transcripts (cDNA sequences) of *B*. *napus* by BLASTN against the *Arabidopsis* Information Resource (TAIR, http://www.arabidopsis.org/Blast/index.jsp) in the present study. Unigenes from TAIR with BLASTN expectation values (E-values) <10^−5^ were considered to be *B*. *napus* homolog genes and were used as rapeseed transcript annotation. For convenience, we analyzed the DETs using the TAIR annotation throughout this study.

### *K*-means clustering analysis

To obtain the expression patterns of DETs during anther development, a *K*-means clustering analysis was performed using the Euclidean Distance based on log_2_ transformed expression values. The clustering process was carried out using GENESIS software (release 1.7.6 beta 1) [23], and *K* choices from 1–50 were evaluated. The *K* choice that yielded the greatest number of distinct expression patters was chosen. Finally, *K* choice 30 was selected for the DETs identified during anther development in rapeseed. To use a smaller *K* value to eliminate clusters with significant patterns, several clusters exhibiting similar patterns (Pearson’s correlation coefficient>0.75) and showing low significant expression alternation between tissues were combined. Finally, 15 dominant patterns (DPs) showing significant gene expression changes during rapeseed anther development were obtained, each DP containing 89 to 4319 transcripts (S1 Table).

### GO term enrichment

A modified ChipEnrich software program [24] was used to further identify enriched GO terms in each DP. GO term enrichment is expressed as a *P*-value calculated from the hypergeometric distribution relative to the GO terms expected to be found in the background. GO terms was considered significantly enriched for *P*<0.001. To visualize GO term enrichment, a heatmap was generated in which the *P* value was log10 transformed and imported into TMeV (http://www.tm4.org/mev.html) [25].

### Identification and visualization of predicted transcriptional modules

To visualize the association of TFs, DNA sequence motifs and enriched GO terms intended to provide insight into the transcriptional programs operating in the anther, ChipEnrich and Cytoscape (version 3.0.1, http://www.cytoscape.org) were used to generate transcriptional modules. ChipEnrich (according to the method of Belmonte *et al*. [24]) generated a <significant.txt> and a <node.txt> file for each DP corresponding to a network file and an attribute file, respectively. These files were then uploaded into Cytoscape, which allows the visualization of the transcriptional network. All network and attribute files can be found in S3 Table.

### Quantitative real-time PCR (RT-qPCR)

Total RNA was isolated using TRIzol reagent from the same samples used for microarray hybridization. For each sample, cDNA was generated from 1 mg of total RNA using the MMLV reverse transcriptase TIANScript RT kit (TIANGEN, China). RT-qPCR was carried out using rapeseed *β-actin* (accession No. AF111812.1) as a reference gene, and the sequences of all primers used in this study are listed in S1 Table. RT-qPCR analysis was performed on a CFX real-time system (Bio-Rad, USA) using platinum SYBR Green qPCR superMix-UDG (invitrogen, USA), with three biological replicates for each sample. The values of the threshold cycle (CT, the fractional cycle number at which the fluorescence passes the fixed threshold) were calculated using CFX manager software and analyzed according to the 2^-ΔΔCt^ method [26].

## Results

### Morphological analysis reveals high metabolic activity during anther development

**Fig 1:**
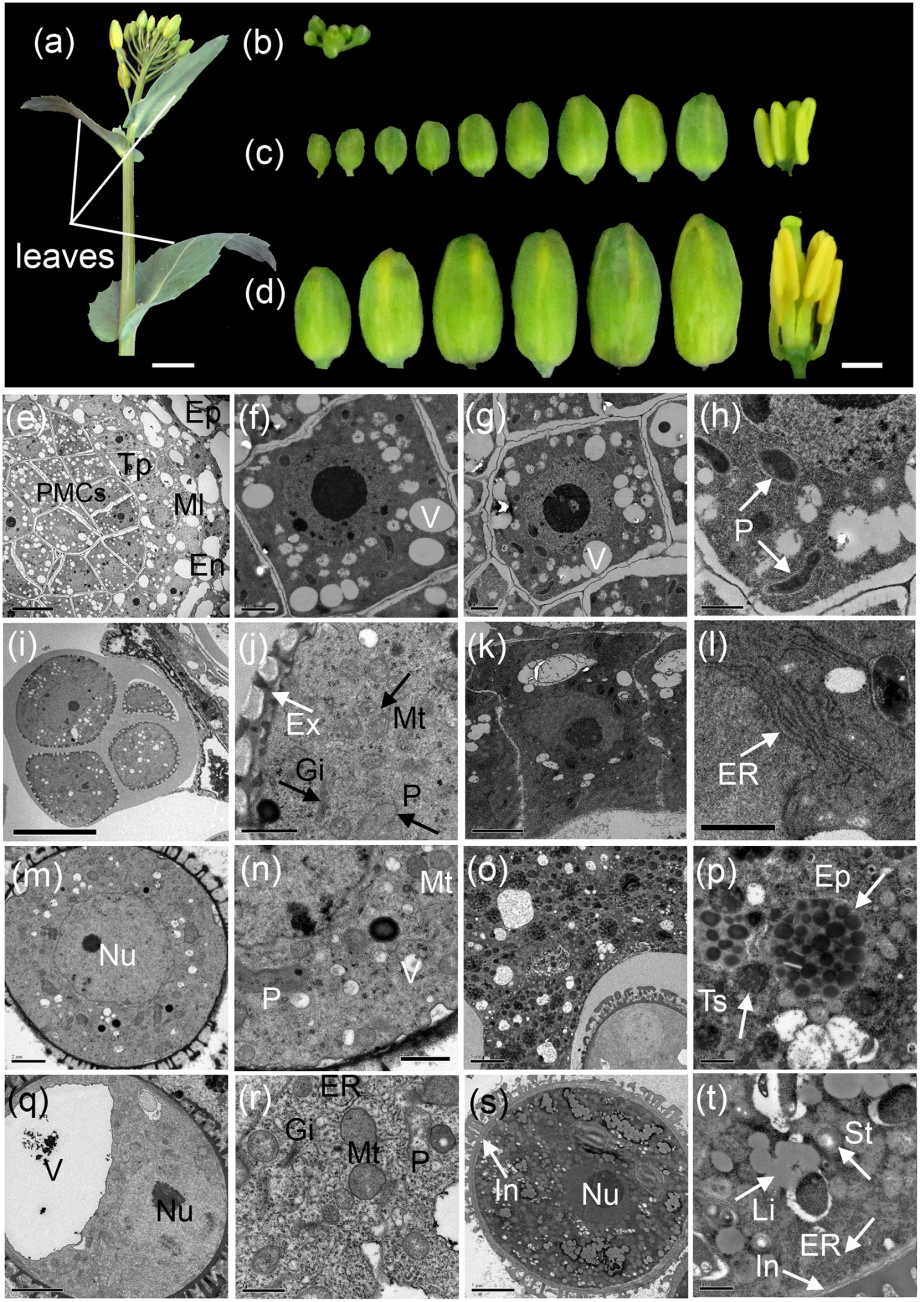
The morphology of samples and ultrastructural features of the anther cells during anther development in rapeseed. (a) The main inflorescence of rapeseed. (b-d) The anther development stages were correlated with the length of the flower buds in rapeseed. Small buds with lengths less than 1 mm (b) contained anthers before and during the pollen mother cell stage; middle buds with lengths of 1–3 mm (c) contained anthers from meiosis to early uninucleate microspore stages; large buds with length larger than 3 mm (d) contained anthers from vacuolated microspore to mature pollen stages. (e-t) Ultrastructural features of the anthers at the pollen mother cell stage (e-h), the meiosis stage (i-l), the early uninucleate microspore stage (m-p), the vacuolated microspore stage (q-r), and the mature pollen stage (s-t). Ep, epidermis; En, endothecium; Ml, middle layer; Tp, tapetum; PMCs, pollen mother cells; P, plastid; Ex, extine; Mt, mitochondrion; Gi, Golgi body; ER, endoplasmic reticulum; Nu, nucleus; V, vacuole; Epl, elaioplast; Ts, tapetosome; In, intine; St, starch granules; Li, lipid bodies. Scale bar in a was 1 cm; scale bars in b-d were1 mm; scale bars in e, i were 10 nm; scale bars in k, o, q, s were 5 nm; scale bars in f, g, j, l, m were 2 nm, scale bars in h, n, r, t were 1 nm.

A similar anther developmental process to that found in *Arabidopsis* occurred in *B*. *napus*, containing a pollen mother cell stage, a tetrad stage, an early uninucleate microspore stage, a vacuolated microspore stage and a mature pollen stage. Previous studies revealed that the rapeseed anther stages correspond to the lengths of flower buds [27]. In this study, we collected leaves and flower buds of different lengths from the main inflorescences in rapeseed plants for transcriptome analysis (Fig 1a, see Materials and methods): leaves (Ls), small buds (SBs) (Fig 1b), the anthers from middle buds (An-MBs) (Fig 1c), and the anthers from large buds (An-LBs) (Fig 1d).

Prior to transcriptome analysis, we examined the ultrastructural features of the anther cells during anther development in rapeseed (Fig 1e–1t). During the pollen mother cell stage, several cell layers appeared in each anther lobe, from the exterior to the interior, the epidermis, the endothecium, the middle layer, the tapetum, and the innermost pollen mother cells (PMCs) (Fig 1e). The epidermis, endothecium and middle layer cells were highly differentiated and exhibited large vacuoles. In contrast, the PMCs (Fig 1f) and the surrounding tapetum (Fig 1g) exhibited high mitotic activity and contained small vacuoles and abundant plastids (Fig 1h, arrow).

During the tetrad stage, PMCs underwent meiosis to form microspores surrounded by a characteristic callose wall (Fig 1i). At this stage, the primexine (Fig 1j, white arrow) and numerous organelles were observed in the cytoplasm, including mitochondria, plastids and Golgi bodies (Fig 1j, black arrow). The tapetum cell cytoplasm was condensed (Fig 1k), and the plastids around the nuclei were surrounded by abundance of endoplasmic reticulum (ER, Fig 1l, arrow). During the early uninucleate microspore stage, the callose wall dissolved, and the microspores were released from the tetrads covered with the exine wall (Fig 1m). The cytoplasm of the microspores was more densely filled with organelles than the previous stage and contained acentral nucleus, many small cytoplasmic vacuoles, and an abundance of mitochondria and plastids (Fig 1n). On the other hand, the tapetum cell wall had dissolved, and the cytoplasm had fused (Fig 1o). In addition, a number of elaioplasts (membrane-bound structures containing numerous lipid droplets, Fig 1p, arrow) and tapetosomes (globular lipid compounds with a high electron density, Fig 1p, arrow) were found in the tapetum.

During the vacuolated microspore stage, the microspores commonly exhibited a single large vacuole, and a nucleus, with a distinct nucleolus, being displaced to one side (Fig 1q). The cytoplasm was rich with organelles, including plastids, dilated ER, lipid bodies, Golgi bodies, and enlarged electron dense mitochondria (Fig 1r). During the mature pollen stage, the microspore contained three nuclei (one vegetative cell and two sperm cells) (Fig 1s). The large vacuole in the microspore was replaced by numerous smaller vacuoles, and the pollen inner wall, which was mainly comprised of pectin, appeared (Fig 1s, arrow). The highly condensed cytoplasm was full of granular organelles, including lipid bodies and plastids containing starch deposits, in addition to an abundance of ER surrounding them (Fig 1t, arrow).

### Transcriptome characteristics of rapeseed anthers at different development stages

We set out to identify the transcriptional programs underlying anther development via the Agilent single channel *Brassica* Oligo Microarray (4×44K, 43,803 probes). As described previously, three independent biological replicates of four tissues (organs) were collected for gene expression analysis, including Ls, SBs, An-MBs and An-LBs (Fig 1a–1d). Correlation coefficients between biological replicates ranged from 0.942 to 0.991, with only one value below 0.95 (S1 Table). This demonstrated that the microarray data obtained in this study were of high quality.

Scatter plot analysis was performed to analyze the extent of transcriptome variation among the three anther developmental stages and the differences between the anther tissues and leaves (S1 Table). These plots indicated that the adjacent developmental stages (i.e., SBs and An-MBs, or An-MBs and An-LBs) showed high correlation values (0.861 and 0.783, respectively), whereas SBs and An-LBs yielded a lower correlation coefficient of 0.674, suggesting that greater alteration of the transcriptome occurred during late anther development stages. Comparing the Ls transcriptome with those of anthers at three developmental stages revealed that SBs and Ls exhibited the highest correlation (*r* = 0.828), which largely caused by the flower envelope of SBs, followed by An-MBs and Ls (*r* = 0.732), then, An-LBs and Ls (*r* = 0.617), indicating the increasing differences in the transcriptome between vegetable and anther tissues over the course of anther development. Notably, global transcriptome alteration between An-MBs and An-LBs (*r* = 0.783) was greater than that between SBs and An-MBs (*r* = 0.861), and even greater than that between Ls and SBs (*r* = 0.828).

**Fig 2:**
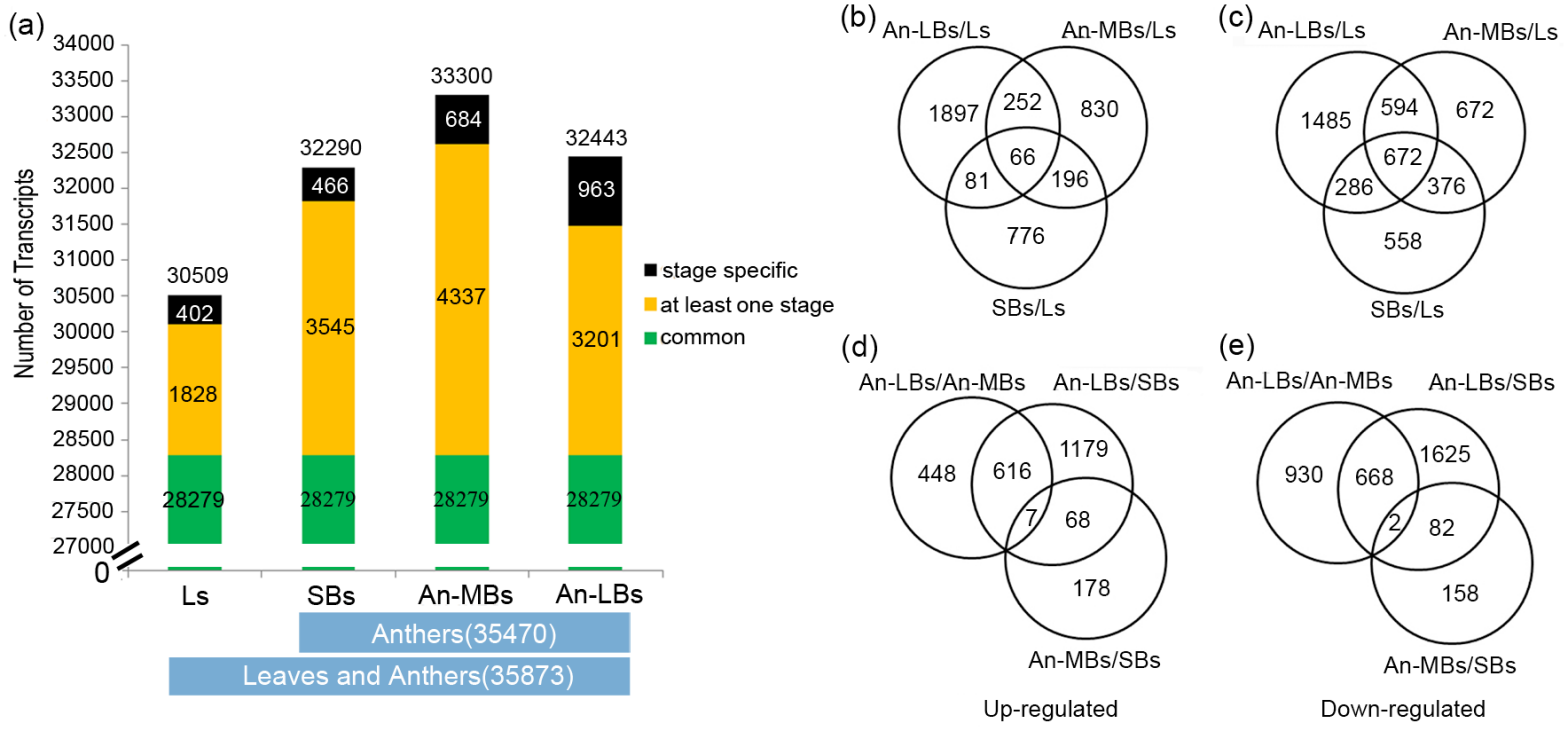
Transcripts detected in leaves (Ls) and anthers at different development stages (a) and Venn diagrams showing the distribution of the differentially expressed transcripts (DETs) between leaves and anthers (b-c) or between different stage anthers (d-e) (b and d, up-regulated; c and e, down-regulated).

To define gene activity in each tissue, the transcripts were filtered according to the calls “present” and “absent” (see Materials and methods) (Fig 2a). An average of 32,136 transcripts (approximately 73% of the total probes on the microarrays) were detected in the tissues of leaves and anthers during different developmental stages, with the lowest number (30,509) in the leaves and the highest (33,300) in the An-MBs. Consequently, 35,873 transcripts were identified as being expressed in at least one tissue, and 35,470 transcripts were expressed in at least one stage of anther development. The total mRNA number from all tissues was not significantly higher than those for each tissue individually, indicating substantial overlap among the four tissues. A total of 28,279 transcripts were simultaneously detected in all four tissues (Fig 2a, in green); in contrast, only 402–963 transcripts were specifically expressed in one tissue (Fig 2a, in black). In addition, the number of stage specific transcripts gradually increased in conjunction with anther differentiation and development. Taken together, An-MBs tissue, which consisted of meiotic to early uninucleate microspores, was found to express the maximum number of genes and amoderate number of stage specific genes. In contrast, An-LBs tissue, which contained vacuolated microspores and mature pollens, showed a comparatively smaller transcriptome and the largest proportion of specific genes.

### Global comparison of gene activity between anthers and vegetative tissue and among different anther stages

To better understand the gene expression dynamics during anther development, Student’s t-test analysis was performed between leaves and anthers, as well as between different stage anthers. In total, the six pairwise comparisons yielded 11,678 differentially expressed transcripts (DETs, Table 1). The transcriptome comparisons between anthers (SBs, An-MBs, or An-LBs) and leaves revealed that a relatively high proportion of DETs were down-regulated as opposed to up-regulated. In addition, the total number of DETs produced in the An-LBs/Ls comparison (5333) was much higher than those of the two previous anther development stage comparisons (An-MBs/Ls, 3658; and SBs/Ls, 3011), which was consistent with the results of the scatter plot analysis (S1 Table). The pairwise transcriptome comparisons of anther tissues revealed that there were more DETs in comparison of An-LBs/An-MBs (2671) and An-LBs/SBs (4247) than An-MBs/SBs (495). This indicated that during late anther development in rapeseed, a large number of genes were reprogrammed. Furthermore, from the SBs to the An-MBs stage, an equivalent number of transcripts were up-(253) or down-regulated (242), most by at least a 10-fold change. However, from the An-MBs to the An-LBs stage, more transcripts (1600) were down-regulated between 2- and 5-fold than up-regulated (1071; most displaying an approximately 10-fold change). These results indicated that a small proportion of genes exhibited significant changes in expression during early anther development. However, during late anther development, fluctuations in gene expression were widespread; a large number of transcripts were adjusted slightly, whereas a small number of transcripts were highly activated.

**Table 1:** Distribution of DETs between leaves and developing anthers or between different developmental anthers.

Fold change	SBs/Ls	An-MBs/Ls	An-LBs/Ls	An-MBs/SBs	An-LBs/An-MBs	An-LBs/SBs
Up-regulated						
2~5	613	797	810	43	260	474
5~10	178	140	316	17	174	261
≥10	328	407	1170	193	637	1135
Subtotal-up	1119	1344	2296	253	1071	1870
Down-regulated						
2~5	768	658	1019	82	1438	1631
5~10	692	610	651	58	128	466
≥10	432	1046	1367	102	34	280
Subtotal-down	1892	2314	3037	242	1600	2377
Total	3011	3658	5333	495	2671	4247

Fold change, the expression level change fold of transcripts between two compared samples.

To further demonstrate the similarities and differences in gene expression alternations during different anther development stages, the DETs identified in each pair-wise tissue comparison were compared (Fig 2b–2e). We found that relatively large numbers of transcripts with altered expression were specific to the corresponding stage transition. Significantly, during An-MBs/SBs and An-LBs/An-MBs stage transitions, rare transcripts (7 up-regulated and 2 down-regulated) were shared between the two stage conversions (Fig 2d and 2e).

### Dominant patterns of gene activity during anther development

**Fig 3:**
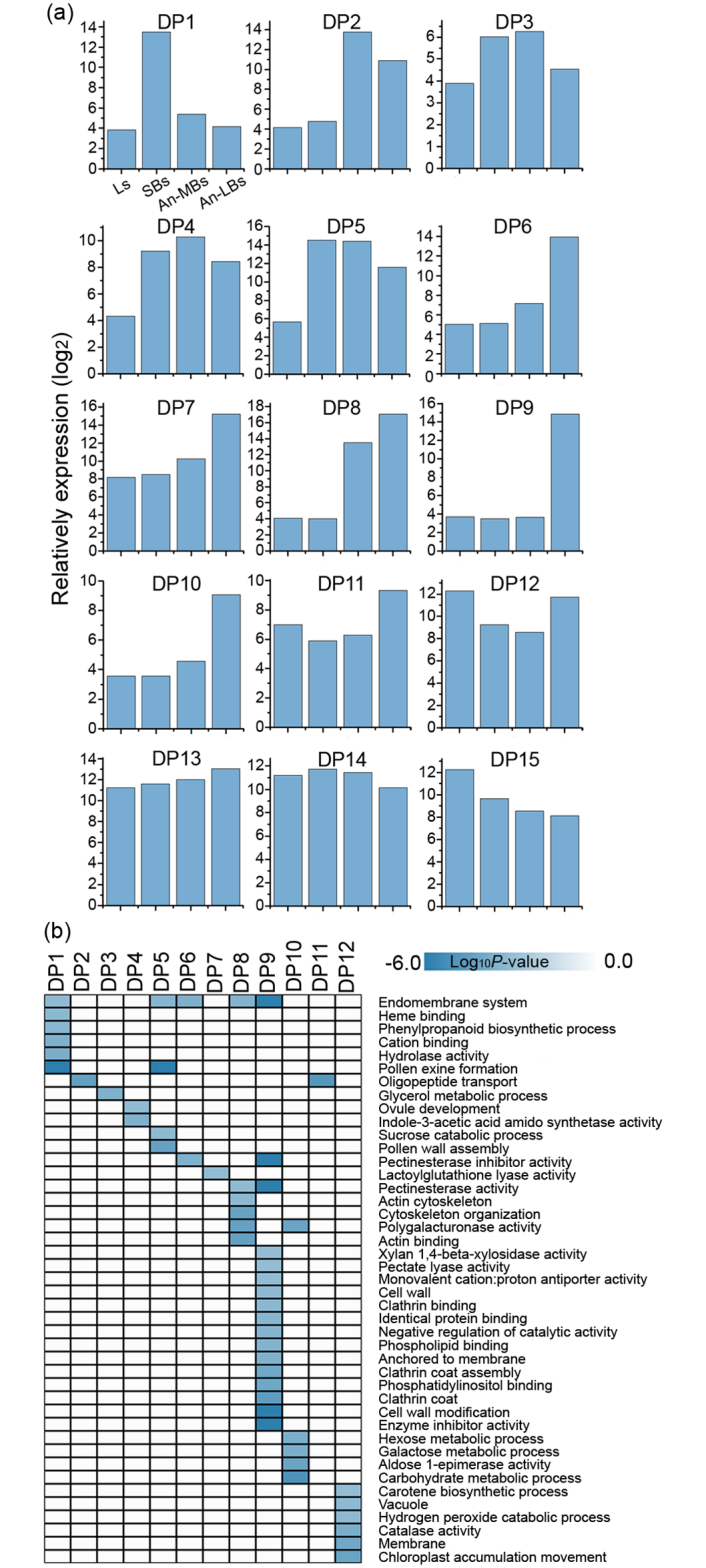
Dominant patterns of transcript expression and biological processes associated with anther development in rapeseed. (a) Fifteen dominant patterns (DPs) of gene activity during rapeseed anther development. DP13, DP14 and DP15 were obtained by combining 4–7 sub-clusters with similar patterns. Detailed information is in S1 Fig. (b) Heatmap of enriched GO terms in DP1~DP12. GO terms were selected at *P*<0.001, with the darker blue color representing a more significant enrichment. The *P*-value was calculated according to a hypothesis test using a cumulative hypergeometric distribution and log_10_ transformed.

To further understand the biological functions underlying anther development in rapeseed, we performed a *k* means clustering analysis of the 11,678 total DETs, and all these DETS were clustered into 15 dominant patterns (DPs) of gene activity with transcript numbers ranging from 89 to 4319 per pattern (S1 Table). Three DPs (DP13, DP14 and DP15) were combined from sub-clusters with significantly similar expression patterns (see Materials and methods, S1 Fig). These 15 DPs revealed different gene expression patterns during anther development (Fig 3a). For example, DP1 and DP2 revealed the preferential expression profiles of SBs and An-MBs, respectively, whereas DP3, DP4 and DP5 contained transcripts that accumulated predominantly in both SBs and An-MBs. Thus, the 705 transcripts contained in DPs 1–5 were associated with early anther development. DP6-DP10 contained transcripts that accumulated predominantly in An-LBs alone, with the exception of DP8, which accumulated in both An-MBs and An-LBs; DP11 and DP12 also exhibited high expression in An-LBs, although these transcripts were also highly expressed in leaves. Then, DP6-DP12 contained 1517 transcripts that were involved in late anther development. In contrast to the significant changes of gene expression, DP13 and DP14 exhibited a slight change in gene expression during anther development. The small difference between DP13 and DP14 was that DP13 showed a consistent increase in gene expression during anther development, whereas DP14 exhibited a slight peak during the SBs and An-MBs stages. Finally, DP15 showed the highest gene activity in Ls and then consistently decreased over the course of anther development. In this study, although all 15 DPs yielded valuable information, DP1-DP12 were the focus of our attention because they exhibited a greater gene expression shift during anther development in rapeseed.

### Dynamic functional alternations during anther development in rapeseed

We next carried out GO term enrichment to predict the cellular components, biological processes, and molecular functions underlying the development of rapeseed anther (Fig 3b, DP1-12; S2 Fig, DP13-15). A large fraction of the genes associated with the endomembrane system were significantly enriched throughout anther development (DP1, *P* = 7.5E-4; DP5, *P* = 4.2E-4; DP6, *P* = 2.3E-4; DP8, *P* = 3.2E-4; DP9, *P* = 4.1E-9). These changes were mirrored by the ultrastructure of the rapeseed anther, which exhibited an abundance of membranous organelles throughout the cytoplasm during anther development (Fig 1). Notably, the ER was rich in the tapetum cells during early anther developmental stages (i.e., the tetrad stage, Fig 1) and also appeared in the cytoplasm of pollen grains during late anther development stages (after the vacuolated microspore), especially in mature pollen (Fig 1t). In addition, oligopeptide transport (DP2, *P* = 3.9E-5; DP11, *P* = 1.6E-5) was also active during both early and late anther development stages.

During early anther development stages (DP1-DP5), transcripts annotated as genes associated with pollen exine formation were detected in SBs and An-MBs tissues (DP1 and DP5, S2 Table), including *LAP3* (*AT3G59530*), *LAP5* (*AT4G34850*), *LAP6* (*AT1G02050*), *CYP704B1* (*AT1G69500*), *MS2* (*AT3G11980*), *ACOS5* (*AT1G62940*), *MEE48* (*AT4G14080*), *CYP703A2* (*AT1G01280*), *NODULIN MTN3 FAMILY PROTEIN* (*AT5G40260*) and *GALACTOSYLTRANSFERASE FAMILY PROTEIN* (*AT1G33430*). Also contributing to the presence of two lipid containing organelles (tapetosomes and elaioplasts, Fig 1p) in tapetum cells during early anther development stages, transcripts annotated as genes encoding enzymes involved in glycerol metabolism were identified in the enriched glycerol metabolism GO term (DP3, *P* = 2.8E-4): *SRG3* (*AT3G02040*), *SDP6* (*AT3G10370*), and *PLC-LIKE PHOSPHODIESTERASES SUPERFAMILY PROTEIN* (*AT5G08030*). In addition, transcripts annotated as genes encoding enzymes involved in sucrose catabolic process were identified (DP5, *P* = 7.4E-4), including *cwINV4 (AT2G36190*) and *PLANT NEUTRAL INVERTASE FAMILY PROTEIN* (*AT4G34860*).

During late anther development stages (DP6-DP12), many GO terms associated with cell wall metabolism were enriched in An-LBs, such as pectinesterase inhibitor activity (DP6, *P* = 2.48E-04; DP9, *P* = 1.17E-06), pectinesterase activity (DP8, *P* = 6.91E-04; DP9, *P* = 6.14E-12), polygalacturonase activity (DP8, *P* = 4.44E-05; DP10, *P* = 6.81E-05), pectatelyase activity (DP9, *P* = 9.54E-04), and cell wall modification (DP9, *P* = 5.35E-07). A large number of transcripts annotated as genes involved in cell wall metabolism were detected at high expression levels in An-LBs (DP6-DP9) and even in both An-MBs and An-LBs (DP8), including *PGA4* (*AT1G02790*), *VGDH2* (*AT3G62170*), *PECTATE LYASE OR LIKE FAMILY PROTEIN* (*AT2G02720*, *AT1G14420*, *AT5G15110*, *AT3G07840*, *AT3G17060*, *AT5G07430*, *AT5G48140*, *AT2G23900*, and *AT5G07420*), and *PLANT INVERTASE/PECTIN METHYLESTERASE INHIBITOR SUPERFAMILY PROTEIN* (*AT3G62180*, *AT1G70540*, *AT2G26450*, *AT3G05610*, *AT5G49180*, *AT1G23350*, *AT5G27870*, *AT1G10770*, *AT2G10970*, *AT2G47050*, and *AT5G50030*). The processes that are highly enriched in An-LBs may be directly associated with or required for pollen intine formation because the changes of these genes are in agreement with the observation that pollen intine does not appear in the rapeseed ultrastructure untill the mature pollen stage (Fig 1s). Interestingly, complementing the high pectin metabolic activity of the anther at the late development stage, the galactose metabolic (DP10, *P* = 1.80E-04) and hexose metabolic (DP10, *P* = 1.99E-04) activity GO terms were also enriched in late stage anthers, including several transcripts annotated as *GALACTOSE MUTAROTASE-LIKE SUPERFAMILY PROTEIN* (*AT3G01260*, *AT4G25900*, *AT5G15140*), with moderate expression level in An-LBs. In addition, An-MBs and An-LBs shared transcripts annotated as genes involved in actin cytoskeleton organization (DP8, *P* = 4.12E-05; 5.38E-05; 6.03E-04): *PRF4* (*AT4G29340*), *PRF5* (*AT2G19770*), *ACT12* (*AT3G46520*), *ADF7* (*AT4G25590*), and *ADF10* (*AT5G52360*). This is in agreement with the occurrence of pollen developmental events involving meiosis and mitosis in An-MBs and An-LBs, respectively. Furthermore, two GO terms (phosphatidylinositol binding and clathrin coat) shared several transcripts annotated as *ENTH/ANTH/VHS SUPERFAMILY PROTEIN* (*AT1G03050*, *AT1G25240*, *AT1G68110*) that were also enriched in DP9 with *p* values of 8.65E-05 and 3.01E-05, respectively. The GO terms enriched in DP13, DP14 and DP15 are shown in S2 Fig and S2 Table.

### Lipid metabolism was associated with early anther development and carbohydrate metabolism was involved in late anther maturation

Our GO enrichment analysis revealed that several GO terms essential for anther development in rapeseed were related to lipid and carbohydrate metabolism, such as pollen extine formation, glycerol metabolism and cell wall metabolism (Fig 3b). This finding was mirrored by our TEM results, which showed that important structures composed of lipids and carbohydrates, such as lipid bodies, and the pollen wall formed during anther development (Fig 1). Thus, we performed a systematic analysis of transcripts annotated as lipid and carbohydrate metabolism related genes in all 15 DPs (Table 2).

**Table 2:** The distribution of lipid and carbohydrate metabolism related transcripts identified in each dominant pattern.

Pathway or Gene Family	DP1	DP2	DP3	DP4	DP5	*DP6*	*DP7*	*DP8*	*DP9*	*DP10*	*DP11*	*DP12*	DP13	DP14	DP15	Total*
**Lipid metabolism**																
Cutin Synthesis & Transport	**0**	**0**	**0**	**0**	**0**	*0*	*0*	*0*	*0*	*0*	*0*	*0*	2	3	0	5 (5)
Eukaryotic Galactolipid & Sulfolipid Synthesis	**0**	**0**	**0**	**0**	**0**	*0*	*0*	*0*	*0*	*0*	*0*	*0*	0	2	2	4 (3)
Eukaryotic Phospholipid Synthesis & Editing	**0**	**0**	**1**	**0**	**0**	*0*	*1*	*1*	*0*	*0*	*1*	*1*	16	5	6	32 (21)
Fatty Acid Elongation & Wax Biosynthesis	**7**	**1**	**4**	**6**	**17**	*0*	*0*	*1*	*0*	*2*	*3*	*1*	17	20	23	102 (68)
Fatty Acid Synthesis	**0**	**0**	**0**	**0**	**0**	*0*	*0*	*0*	*0*	*0*	*1*	*0*	14	12	8	35 (26)
Lipid Trafficking	**0**	**0**	**0**	**0**	**0**	*0*	*0*	*0*	*0*	*0*	*0*	*0*	2	0	1	3 (3)
Mitochondrial Fatty Acid &Lipoic Acid Synthesis	**0**	**0**	**0**	**0**	**0**	*0*	*0*	*0*	*0*	*0*	*0*	*0*	3	6	2	11 (6)
Mitochondrial Phospholipid Synthesis	**0**	**0**	**1**	**0**	**0**	*0*	*0*	*0*	*0*	*0*	*0*	*0*	0	0	2	3 (2)
Oxylipin Metabolism	**0**	**0**	**0**	**2**	**0**	*1*	*1*	*0*	*1*	*1*	*1*	*0*	4	3	2	16 (14)
Phospholipid Signaling	**0**	**0**	**0**	**0**	**0**	*2*	*1*	*0*	*4*	*0*	*1*	*0*	17	11	4	40 (28)
Prokaryotic Galactolipid, Sulfolipid, & Phospholipid Synthesis	**0**	**0**	**0**	**0**	**0**	*0*	*0*	*0*	*0*	*0*	*1*	*0*	6	11	19	37 (22)
Sphingolipid Biosynthesis	**0**	**0**	**0**	**0**	**0**	*0*	*1*	*0*	*3*	*1*	*2*	*0*	12	0	0	19 (14)
Suberin Synthesis & Transport	**1**	**0**	**1**	**0**	**0**	*0*	*0*	*0*	*0*	*0*	*0*	*0*	0	0	1	3 (3)
Triacylglycerol & Fatty Acid Degradation	**0**	**1**	**1**	**0**	**0**	*0*	*0*	*0*	*0*	*0*	*1*	*5*	26	2	4	40 (30)
Triacylglycerol Biosynthesis	**0**	**6**	**1**	**0**	**1**	*0*	*0*	*4*	*1*	*0*	*1*	*1*	15	8	4	42 (30)
Unknown	**0**	**0**	**3**	**0**	**0**	*0*	*1*	*0*	*2*	*0*	*1*	*0*	8	6	10	31 (23)
Total*	**8 (5)**	**8 (5)**	**12 (11)**	**8 (7)**	**18 (9)**	*3 (2)*	*5 (5)*	*6 (5)*	*11 (8)*	*4(4)*	*13 (13)*	*8 (7)*	142 (95)	89 (64)	88 (58)	423 (298)
**Carbohydrate metabolism**																
Glycoside Hydrolase Family (GH)	**7**	**2**	**2**	**2**	**5**	*8*	*4*	*21*	*9*	*12*	*7*	*7*	40	30	38	194 (141)
Glycosyl Transferase Family (GT)	**0**	**1**	**5**	**5**	**1**	*4*	*5*	*2*	*4*	*8*	*11*	*8*	74	41	51	220 (177)
Polysaccharide Lyase Family (PL)	**0**	**0**	**0**	**0**	**0**	*0*	*0*	*2*	*6*	*1*	*0*	*1*	3	2	2	17 (10)
Carbohydrate Esterase Family (CE)	**0**	**1**	**0**	**1**	**0**	*1*	*2*	*15*	*12*	*0*	*1*	*3*	1	2	2	41 (25)
Auxiliary Activity Family (AA)	**0**	**0**	**0**	**0**	**0**	*0*	*1*	*0*	*0*	*1*	*0*	*0*	0	0	0	2 (2)
Carbohydrate-Binding Module Family (CBM)	**3**	**0**	**0**	**0**	**3**	*0*	*0*	*0*	*0*	*0*	*0*	*0*	2	19	12	39 (31)
Total*	**10 (6)**	**4 (4)**	**7 (7)**	**8 (8)**	**9 (5)**	*13 (11)*	*12 (11)*	*40 (14)*	*31 (20)*	*22 (21)*	*19 (17)*	*19 (17)*	120 (83)	94 (68)	105 (77)	493 (369)

Dominant patterns at early (DP1-5,) and late (DP6-DP12) anther development stages were in bold and italic, respectively. The relatively enriched pathways or gene families were highlighted in underline.

*: the number in bracket after was the total unique AGIs identified in each DP corresponding to lipid or carbohydrate metabolism.

**Fig 4:**
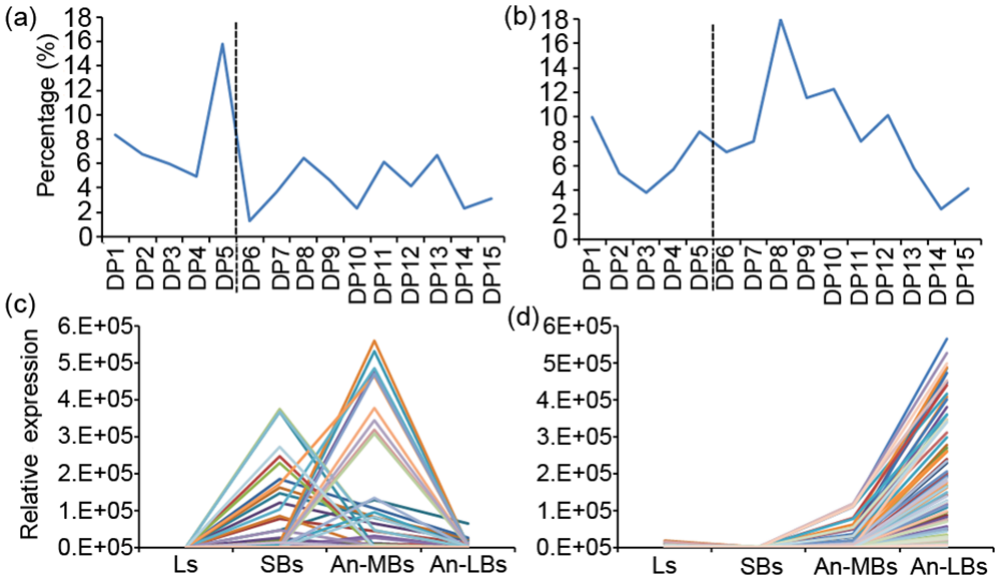
The distribution of lipid and carbohydrate metabolism related genes in each dominant pattern (DP) and the expression profiles of the corresponding transcripts during rapeseed anther development. (a-b) The percentage of unique AGIs with functions related to lipid (a) or carbohydrate (b) metabolism to total unique AGIs annotated in each DP was graphed, showing a relatively high percentage of lipid metabolism genes in DP1-DP5 and a high percentage of carbohydrate metabolism genes in DP6-DP12. (c-d) The expression profiles representing transcript levels of genes involved in lipid metabolism in DP1-DP5 (c) and carbohydrate metabolism (d) in DP6-DP12.

In total, 423 transcripts annotated as genes involved in lipid metabolism exhibited dynamic expression profiles throughout anther development in rapeseed (Table 2, S3 Table). They were distributed among 15 lipid metabolism pathways, with the very long chain fatty acid synthesis (*Fatty Acid Elongation & Wax Biosynthesis*) pathway containing the most transcripts (102), followed by triacylglycerol metabolism (*Triacylglycerol Biosynthesis*, 42 and *Triacylglycerol & Fatty Acid Degradation*, 40) and *Phospholipid Signaling* (40). The percentage of unique AGI genes related to lipid metabolism out of the total unique AGIs annotated in each DP indicated that a relatively high percentage of lipid metabolism genes was clustered in DP1-DP5 (Fig 4a), with a preponderance in DP5. Furthermore, these enriched genes were dominant in *Fatty Acid Elongation & Wax Biosynthesis* (Table 2), including *MS2* (*AT3G11980*), *lipid transfer proteins* (*LTP6*, *AT3G08770*; *LTP12*, *AT3G51590*), and *3-ketoacyl-CoA synthase* (*KCS7*, *AT1G71160*; *KCS15*, *AT3G52160*; *KCS19*, *AT5G04530*; *KCS21*, *AT5G49070*), which may be involved in pollen exine wall formation during early anther developmental stages (S3 Table). Another lipid metabolism related gene population was clustered in the *Triacylglycerol Biosynthesis* pathway, including genes such as *Oleosin family protein* (*AT5G07600*, *AT5G07571*, *AT3G27660*), *Glycine rich protein 19* (*GRP19*, *AT5G07550*), and *Plant specific transcription factor YABBY family protein* (*AT1G69180*), which may be related to lipid body formation in tapetum cells (S3 Table). The lipid related genes enriched in DP1-DP5 were highly expressed in SBs and/or An-MBs (Fig 4c).

Furthermore, 493 transcripts were annotated as genes involved in carbohydrate metabolism exhibiting dynamic expression profiles throughout anther development (Table 2, S3 Table). These genes were clustered into six gene families. The two largest families were *Glycoside Hydrolase Family* (*GH*) and *Glycosyl Transferase Family* (*GT*), containing 194 and 220 transcripts, respectively. The percentage of unique AGI genes related to carbohydrate metabolism of the total unique AGIs annotated in each DP was relatively high in DP6-DP12 (Fig 4b), especially in DP8. In addition, these genes were mainly found in the *Glycoside Hydrolase Family* (*GH*), the *Glycosyl Transferase Family* (*GT*), and the *Carbohydrate Esterase Family* (*CE*), including a large number of invertase or pectin lyase related genes (S3 Table) that may be required for pollen intine wall formation during late anther developmental stages. Coincidently, the carbohydrate related genes enriched in DP6-DP12 were highly expressed in An-LBs (Fig 4d).

Complementary to these stage preferentially expressed genes, a large number of lipid and carbohydrate metabolic genes were identified in DP13 (increased slowly over development) and DP14 (constant expression in each tissue, with a slight peak in SBs and An-MBs). These lipid and carbohydrate metabolism related genes may function fundamentally in all tissues to keep normal growth and development.

### Predictive regulatory networks reveal that the *AG* regulator motif related genes maybe involved in pollen wall formation

**Fig 5:**
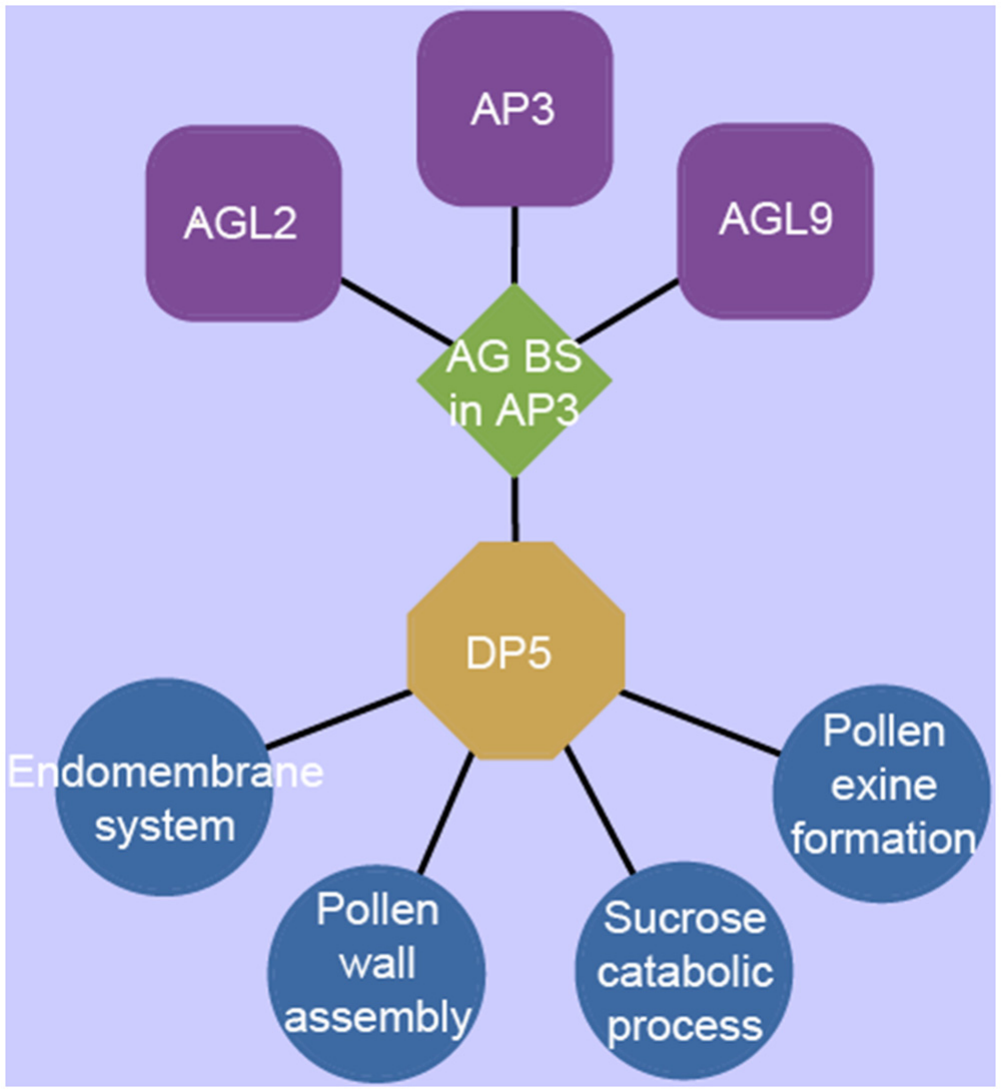
Predicted AG transcriptional module during early anther development stages in rapeseed. GO terms (blue circles) were enriched in DP5 (yellow octagon). The AG binding site motif (green diamond) is also enriched in DP5 and is predicted to be a target of three transcription factors (purple squares). Enrichment is at *P*<0.001 (hypergeometric distribution).

In this study, we identified 478 DETs annotated as transcription factors (TFs) dispersing among 43 distinct TF families with different dominant expression patterns (S3 Table). The generation of predictive transcriptional modules can be used to identify specific regulators operating on the transcriptional circuitry responsible for coordinating developmental processes [24]. Thus, we generated a predictive transcriptional module that links transcription factors with their potential target genes. This strategy associates DNA sequence motifs that are significantly enriched in the upstream regions of coexpressed genes (*P*<0.001, hypergenmetric distribution) with coexpressed transcription factors that are known or predicted to bind the overrepresented motifs. In this study, the predictive transcriptional module analysis for anther development in rapeseed was performed based on AGI genes annotated in *Arabidopsis*. A predictive transcriptional module for DP5 (Fig 5, S2 Table) identified three transcription factors interacting with the *AG* binding site motif as follows: *AGL2 (AT5G15800)*, *AP3 (AT3G54340)*, and *AGL9 (AT1G24260)*, all of which are *K-box* region and *MADS-box* transcription factors. DP5 was enriched for the GO terms: pollen wall assembly (4.4E-05), pollen exine formation (2.5E-10), the endomembrane system (4.2E-04), and sucrose catabolism (7.4E-04) (S3 Table). This suggests that the *AG* regulator motif related genes maybe involved in pollen wall formation.

### Validation of specific expression profiles by RT-qPCR

The microarray data were confirmed in our previous report by RT-qPCR [19] reactions for 62 randomly selected genes, revealing a high degree of concordance (R^2^ = 0.8775) between the microarray and RT-qPCR results. In this study, we selected eight transcripts in different DPs exhibiting preferential expression in one or more stages for RT-qPCR analysis (S4 Fig), including two transcripts (*A_46_P165304*, *A_46_P119219*) from DP1, one transcript (*A_46_P172054*) from DP2, one transcript (*A_46_P313520*) from DP7, two transcripts (*A_46_P074236*, *A_46_P132729*) from DP8, one transcript (*A_46_P133334*) from DP10 and one transcript (*A_46_P311390*) from DP13. The overall gene expression identified by our microarray experiments exhibited a high degree of similarity with the results obtained by RT-qPCR, with a correlation co-efficient (r) greater than 0.9, thereby indicating the reliability and robustness of the microarray data.

## Discussion

### Rapeseed anther ultrastructure analysis reveals numerous organelles abundant with metabolic materials appearing during the anther development stage

In plants, successful development of the male reproductive organ, the anther, requires a sequence of developmental events [28–31]. To examine this process in detail, previous studies have staged anther development in tobacco [32], *Arabidopsis* [4] and rice [33]. In *Arabidopsis*, anther development has been divided into 14 stages based on morphological features [4,30] according to the key events happening in anther cells. Overall, the developmental events are highly similar between rapeseed and *Arabidopsis*. We therefore characterized the rapeseed anther developmental process according to the development stages of *Arabidopsis*, with a focus on the pollen mother cell stage, the tetrad stage, the early uninucleate microspore stage, the vacuolated microspore stage, and the mature pollen stage.

Ultrastructural analysis revealed that numerous organelles abundant with metabolic materials, such as elaioplast, tapetosomes, plastids (containing starch deposits) etc. appeared in microspores and tapetum cells throughout the anther developmental stages of rapeseed. At the pollen mother cell stage, the difference between PMCs and tapetum cells was not significant, and many plastids were observed in the cytoplasm of both type of cells (Fig 1h). Afterward, PMCs enter meiosis to form tetrads, whereas tapetum cells became secretory, exhibiting abundant ER and condensed cytoplasm (Fig 1k and 1l). Over the course of anther development, many membranous organelles appeared in microspores and tapetum cells, including the ER, Golgi body, plastids and mitochondria, indicating high metabolic activity in microspores and tapetum cells throughout anther development (Fig 1). In particular, at the early uninucleate microspore stage, two specialized organelles, elaioplasts and tapetosomes, were observed in tapetum cells in rapeseed anther (Fig 1o and 1p). These organelles are abundant in tapetum cells during the active stage of pollen maturation in *Brassicaceae* species, including *Arabidopsis* [34]. Previous studies have reported that elaioplasts are specialized plastids derived from proplastids [35,36] which contain few internal membranes that was packed with globules of steryl esters enclosed by structural proteins [37]. Meanwhile, lipid-rich tapetosomes are composed of oleosin-coated oil droplets and vesicles, both of which are assembled in and then detached from the ER [38,39]. This lipid droplet structure is similar to that of a seed oil body, containing triacylglycerols (TAGs) enclosed by amphipathic structural proteins termed oleosins, as well as, presumably, phospholipids (PLs). After tapetum cell degradation, both tapetosomes and elaioplasts were deposited onto the maturing pollen to form the pollen coat, which is the outermost layer of the pollen wall [34,38–40]. Pollen wall formation is an essential metabolic process throughout pollen development and consists of three layers as follows: an inner pectocellulosic intine, an outer sporopollenin based exine and a lipid- and protein-rich pollen coat in the crevices of exine. As early as the tetrad stage, when microspores are packed with callose, the individual microspores initiate to develop the exine. Meanwhile, the pectocellulosic intine and pollen coat were formed after the microspores were released from the callose [28,41]. At the mature pollen stage, the microspores enter mitosis to produce the three-cell pollen grains that are compacted with various materials in the cytoplasm reserved for germination, including lipids and starch (Fig 1s and 1t,).

Therefore, we conclude that the main processes contributing to pollen development and function are cellular meiosis and mitosis, pollen wall formation, material accumulation for germination, and perhaps other assistant metabolic processes, all of which are the foundation of pollen to perform fertilization ability.

### Changes of gene expression during anther development are similar between rapeseed and other plant species

Studies profiling the developmental transcriptome of the male gametophyte have been performed in different plant species, including *Arabidopsis*, rice and maize. Honys and Twell [13] performed a developmental transcriptome analysis using density gradient centrifugation to separate the four stages, from microspore to mature pollen as follows: uninucleate microspore (UNM), bicellular pollen (BCP), tricellular pollen (TCP), and mature pollen grain (MPG). In total, 13,997 genes were expressed in the male gametophyte, with the transcriptome size decreasing progressively from 11,565 genes in UNM to 7,235 genes in MGP. In contrast, the percentage of pollen specific genes increased from 6.9% in UNM to 8.6% in MPG, reflecting the differentiation and functional specialization of mature pollen. Pairwise comparisons showed that the expression profiles of UNM and BCP (*R* = 0.96) and TCP and MPG (*R* = 0.86) were well correlated; however, the profiles were less similar for the BCP and TCP (*R* = 0.54) stages. This trend reflects the bicellular stage as transition between the early proliferative and late differentiation phase. Similar changes were also observed during anther development in rice [18] and maize [42].

In this study, we performed a transcriptome analysis using an Agilent *Brassica* Oligo Microarray (4×44K) of three stages of rapeseed anther, including SBs (containing anthers before and during the pollen mother cell stage), An-MBs (from meiosis to the early uninucleate microspore stage) and An-LBs (vacuolated microspore to mature pollen stages), which covered the entirety of anther development in rapeseed. These results indicated that 35,470 transcripts were expressed in at least one of the anther development stages from the pollen mother cell stage to the mature pollen stage, with the highest transcript number in An-MBs (33300) and the lowest in SBs (32290) (Fig 2a). This result is consistent with those of previous studies in *Arabidopsis* and rice, which revealed the most complex transcriptomes during meiosis. However, the number of stage specific transcripts increased progressively from SBs (466) to An-LBs (964), indicating that differentiation occurred during anther development and the mature pollen exhibited the most specialization, as indicated in previous reports. Overall, changes in the transcriptome were also inferred by the decreased correlation coefficient from the early (SBs and An-MBs; *r* = 0.861) to the late (An-MBs and An-LBs; *r* = 0.783) stage of development.

Furthermore, a global comparison of gene activity in developing anthers revealed that more genes were expressed during the late stage transformation from An-MBs to An-LBs (1071 up-regulated and 1600 down-regulated) compared with the transition from SBs to An-MBs (253 up-regulated and 242 down-regulated). The transformation from An-MBs to An-LBs exhibited the greatest number of genes down-regulated by approximately 2~5-fold and a large number of genes up-regulated by more than 10-fold. In contrast, the transformation from SBs to An-MBs contained the greatest number of genes that were up- or down- regulated by more than 10-fold. These results indicated that there was a gene expression alteration of great magnitude during early anther development from the pollen mother cell stage to the tetrad stage, although the number of regulated genes was relatively small. However, during the late anther development stages from uninucleate microspore to mature pollen stage, a large number of genes changed their expression profile, with a proportion of genes being significantly up-regulated and a large number of genes being programly repressed. Therefore, this study suggests that changes in gene expression occurred throughout anther development to cooperate metabolism in anther and the formation of cellular structures, with the latter stages exhibiting greater changes than early stages.

### Functional alteration corroborates ultrastructural features and exhibits a shift from lipid to carbohydrate metabolism during anther development

Our data provide strong evidence for the occurrence of a variety of biological processes throughout anther development. The changes in gene expression were in agreement with the ultrastructural features observed during anther development.

During early stages of anther development, the endomembrane system and pollen exine formation appear to be highly active in the anther (Fig 3b, DP1-DP5), which is consistent with the ultrastructure observations shown in Fig 1i–1l. The presence of transcripts annotated as genes involved in the endomembrane system suggests a high degree of anabolic metabolism in the anther, which may provide a substrate for pollen exine formation. Furthermore, transcripts annotated as genes associated with glycerol metabolism and sucrose catabolic processes were also enriched in early stage anthers (Fig 3b, DP3 and DP5). Indeed, triacylglycerol (TAG) was one of the main components of the tapetosome, which is the typical tapetum organelle in crucifers and was also observed in this study (Fig 1o and 1p). We presumed that the sugars produced through invertase mediated sucrose catabolism during the early stage of anther development may serve as substrates for TAG anabolic metabolism in the tapetosome.

During the maturation of the anther, several GO terms associated with cell wall metabolism were enriched (Fig 3b), including pectinesterase inhibitor activity, pectinesterase activity, polygalacturonase activity, pectatelyase activity, and cell wall modification. This high level of pectin metabolic activity may be directly associated with or required for pollen intine formation. Since the main component of pollen intine is pectin and it is initiated to form during late anther developmental stages after microspores are released from the callose [28]. In this study, we did not observe the pollen intine wall, which was the thickening of the cytoplasmic side of the pollen wall, until the mature pollen stage (Fig 1s and 1t). Furthermore, galactose and hexose metabolism were also active during the anther maturation stage (Fig 3b). We speculated that they may be involved in pollen starch synthesis because mature pollen rich in starch is a marker of pollen maturity (Fig 3t), which may serve as an energy reserve for germination [11,43].

Taken together, there appears to be a functional switch from lipid preferential metabolism to carbohydrate dominant metabolism from the early to the late stages of anther development. Early lipid metabolism pathways were mainly associated with the very long chain fatty acid synthesis (*Fatty Acid Elongation & Wax Biosynthesis*) pathway and the triacylglycerol metabolism pathway (*Triacylglycerol Biosynthesis* and *Triacylglycerol & Fatty Acid Degradation*) (Table 2). We inferred that the former was involved in pollen exine formation and that the latter was associated with tapetosome formation. The late carbohydrate metabolism gene families were the *Glycoside Hydrolase Family* (*GH*), the *Carbohydrate Esterase Family* (*CE*) and the *Glycosyl Transferase Family* (*GT*), including a large number of invertase and pectin lyase related genes (S3 Table) that maybe required for pollen intine wall formation. In plants, triacylglycerol was considered to be a form of carbon storage, and its synthesis occurred primarily in the embryo and other zygotic tissues of the seed [44,45]. In this study, the tapetosomes in anther tapetum cells also accumulated TAGs, combined with steryl esters and oleosins. However, during late stages of anther development, the latter two components (steryl esters and oleosins) are discharged to the locule and deposited onto maturing pollen to form the pollen coat [38]. Until now, no studies report where the TAGs had localized. Are the remaining TAGs in tapetosomes used for carbohydrate metabolism, including pectin and starch synthesis, during the pollen mature stage? It remains to be further studied.

### Predictive metabolic regulatory network

Previous studies have identified many TFs during pollen development, some of them were male gametophyte specific [13,18,42]. In this study, we identified 478 transcripts annotated as TF genes with differential expression patterns (S3 Table). Interestingly, among them, several annotated TFs were reported to control pollen wall formation during *Arabidopsis* anther or pollen development, such as *AMS* and *AtBZIP34*. *AMS*, which encodes a bHLH transcription factor, regulates the expression of a number of genes involved in various biological activities, particularly those associated with metabolism and the deposition of the pollen wall [46,47], whereas *AtBZIP34* has been reported to control pollen wall patterning and to affect several metabolic pathways [48]. Similarly, *MS1*, which encodes a putative PHD finger (Plant Homeo Domain) protein [49–52], was also proposed to regulate the expression of sporophytic genes necessary for pollen extine formation. Considering that the metabolic processes occurring during anther development could be regulated by TFs, we performed a predictive transcriptional module analysis within gene populations exhibiting different dominant patterns, resulting in the identification of a transcriptional module in DP5. Five transcripts annotated as three *MADS-box* transcription factors (*AP3*, *AGL2*, and *AGL9*) and the corresponding binding motifs in the upstream regions of co-expressed genes were enriched. The co-expressed genes were predicted to function in pollen wall assembly, pollen exine formation, the endomembrane system, and sucrose catabolism, indicating that these TFs may also be involved in pollen wall formation. Although previous studies on these three TFs were mainly focused on early flower organ specification [53,54], one recent report has noted that the flowers of the *ap3* mutant had a distinct glycerolipid composition compared with that of the wild type [55]. In this study, the consistently high expression levels of the three TFs throughout anther development suggests that they may also be involved in the regulation of metabolism required for pollen extine formation during early anther development (S3 Table).

## Conclusion

This study provides a comprehensive and systematic analysis of metabolic processes during rapeseed anther development using anatomy and genome-wide transcriptome analysis. Our results indicated that lipid and carbohydrate metabolism are active throughout anther development and that gene expression shifted from lipid preferential to carbohydrate dominant metabolism occurred between early and late anther development. The main events involved in anther or pollen development were pollen wall formation, cell meiosis and mitosis, and energy storage for germination, which form the foundation for the ability of pollen to deliver male gametes to the embryo sac for double fertilization. Transcription factors co-expressed with metabolic genes were identified and predicted to regulate these events. This analysis furthers our understanding of the dynamic gene expression programming underlying anther development, especially with regard to metabolism during pollen development. However, the metabolic processes regulatory networks, and the cross talk between different metabolic substrates remain to be characterized during anther development,.

## Supporting Information

S1 Fig The expression patterns of sub-clusters in DP13, DP14 and DP15. Four, seven, and seven sub-clusters in DP13, DP14 and DP15, respectively.
(TIF)

S2 Fig Heatmap of enriched GO terms in DP13, DP14 and DP15. GO terms were selected at *P*<0.001, with the darker blue color representing a greater significant enrichment. The *P*-value was calculated according to a hypothesis test using cumulative hypergeometric distribution and log_10_ transformed.
(TIF)

S3 Fig The predicted transcriptional module in DP14 (yellow octagon). Two motifs (green diamond) and many GO terms (blue circles) were enriched. Detailed information was deposited in S3 Table.
(TIF)

S4 Fig The RT-qPCR analysis of eight genes showing a high correlation with the microarray data. Three biological replicates were taken for both RT-qPCR and microarray analysis. The Y axis represents normalized log_2_ transformed expression values obtained using microarray analysis and RT-qPCR, respectively. The RT-qPCR data have been scaled such that the minimum expression value of RT-qPCR equals that of the minimum value of the microarray to ease profile matching. The correlation coefficient (r) between the two expression profiles is also indicated. Expression of *B*. *napus β-actin* (accession no. AF111812.1) was used as an internal control to normalize the RT-qPCR data.
(TIF)

S1 Table Correlation coefficients of microarrays, differentially expressed transcript list, transcript list of each dominant pattern (DP), RT-qPCR primer list. (XLSX)

S2 Table Lists of GO terms in each dominant pattern (DP) of gene activity during rapeseed anther development and DNA sequence motifs in DP5. (XLSX)

S3 Table Lipid and carbohydrate metabolism related gene list, TFs, network and attribute files in DP5 and DP14 required for network visualization. (XLSX)
