# Supplementary figures and images for: Global Dynamic Transcriptome Programming of Rapeseed (Brassica napus L.) Anther at Different Development Stages
Source: PLoS One. 2016 May 3;11(5):e0154039. doi: 10.1371/journal.pone.0154039 (PMC4854403; doi:10.1371/journal.pone.0154039)

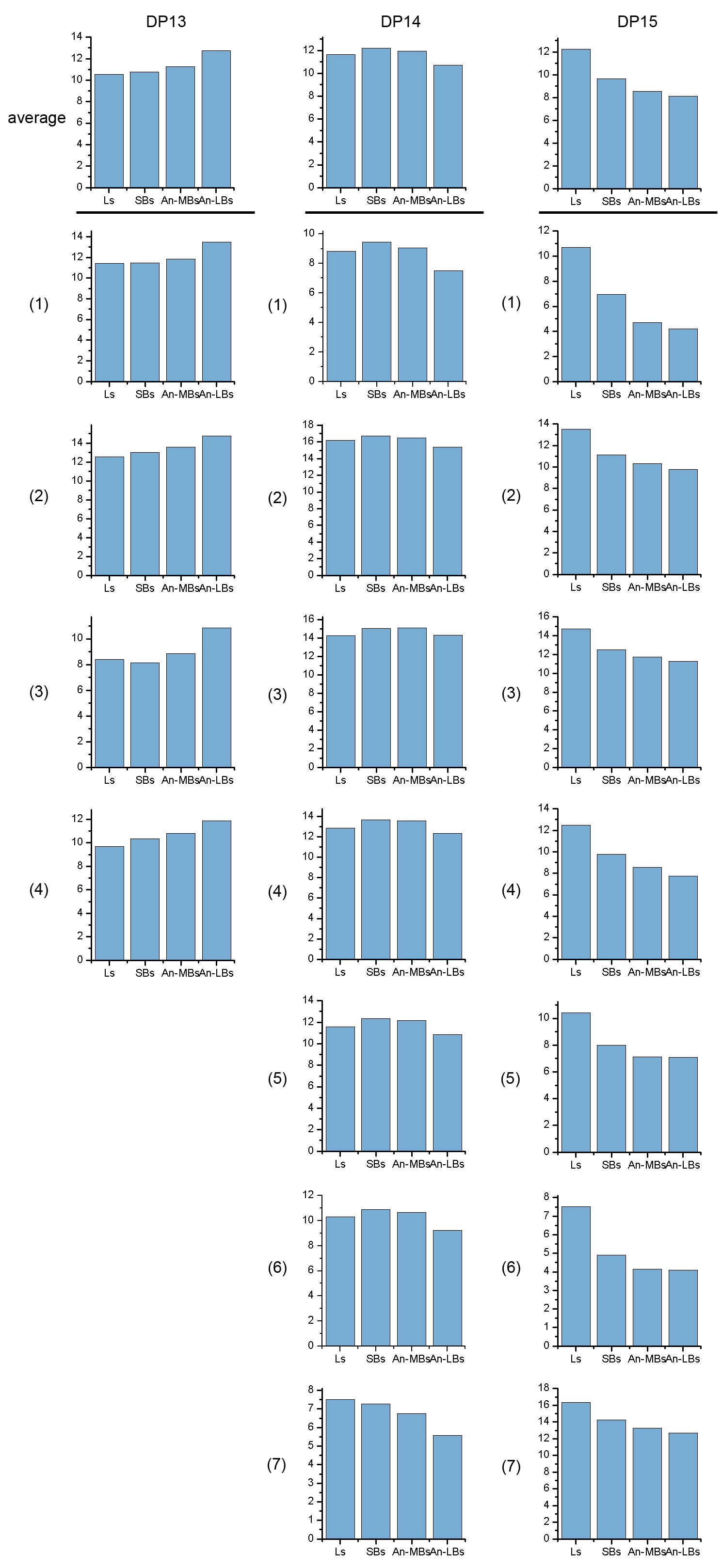

Supplement: S1 Fig — Four, seven, and seven sub-clusters in DP13, DP14 and DP15, respectively. (TIF) [file pone.0154039.s001.tif]

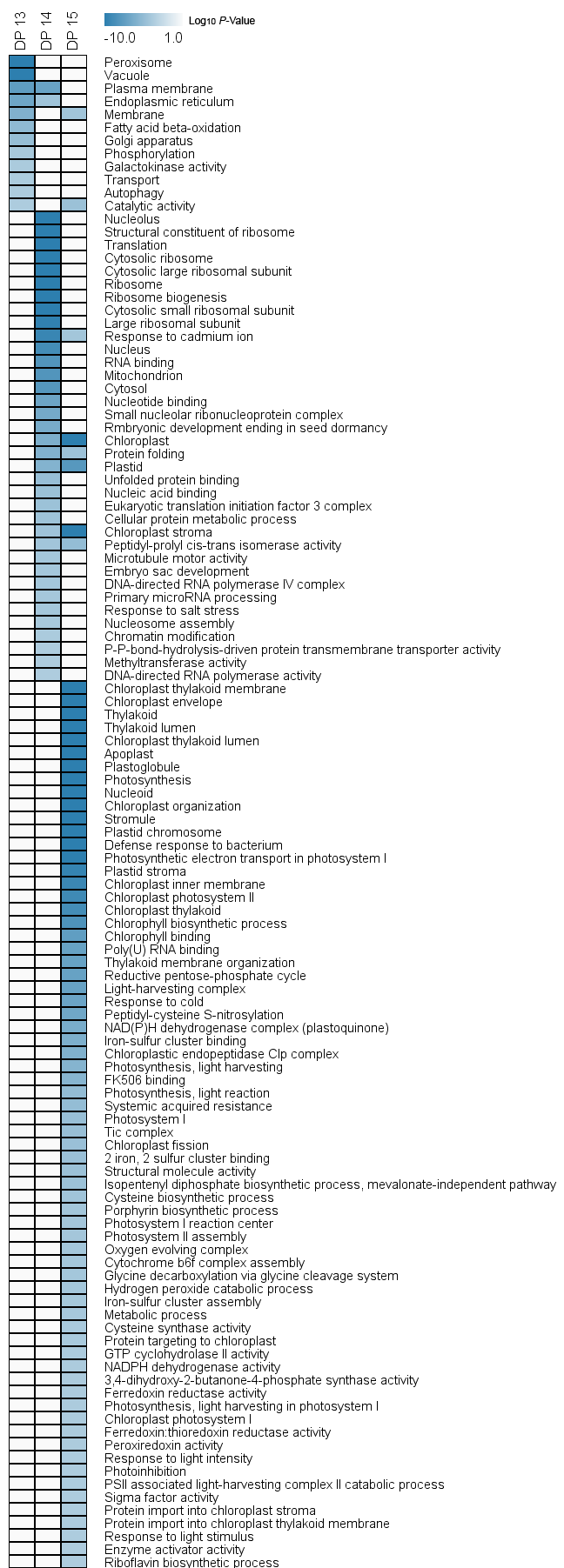

Supplement: S2 Fig — GO terms were selected at P<0.001, with the darker blue color representing a greater significant enrichment. The P-value was calculated according to a hypothesis test using cumulative hypergeometric distribution and log10 transformed. (TIF) [file pone.0154039.s002.tif]

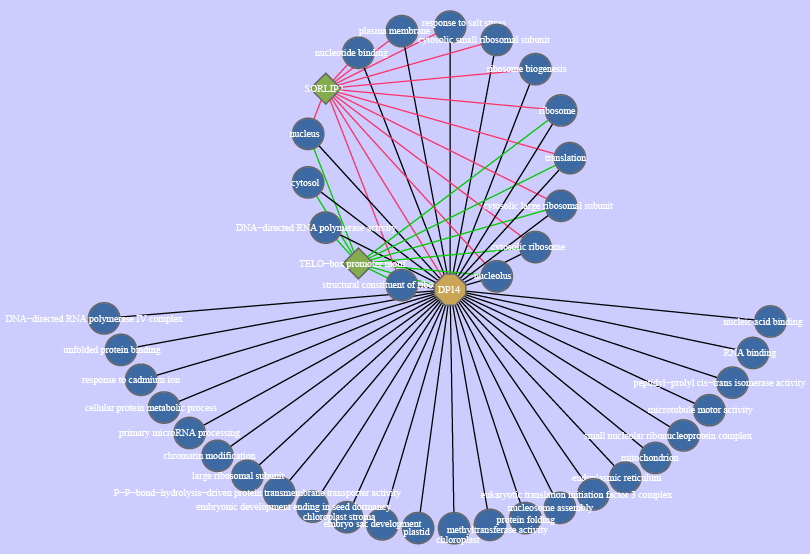

Supplement: S3 Fig — Two motifs (green diamond) and many GO terms (blue circles) were enriched. Detailed information was deposited in S3 Table. (TIF) [file pone.0154039.s003.tif]

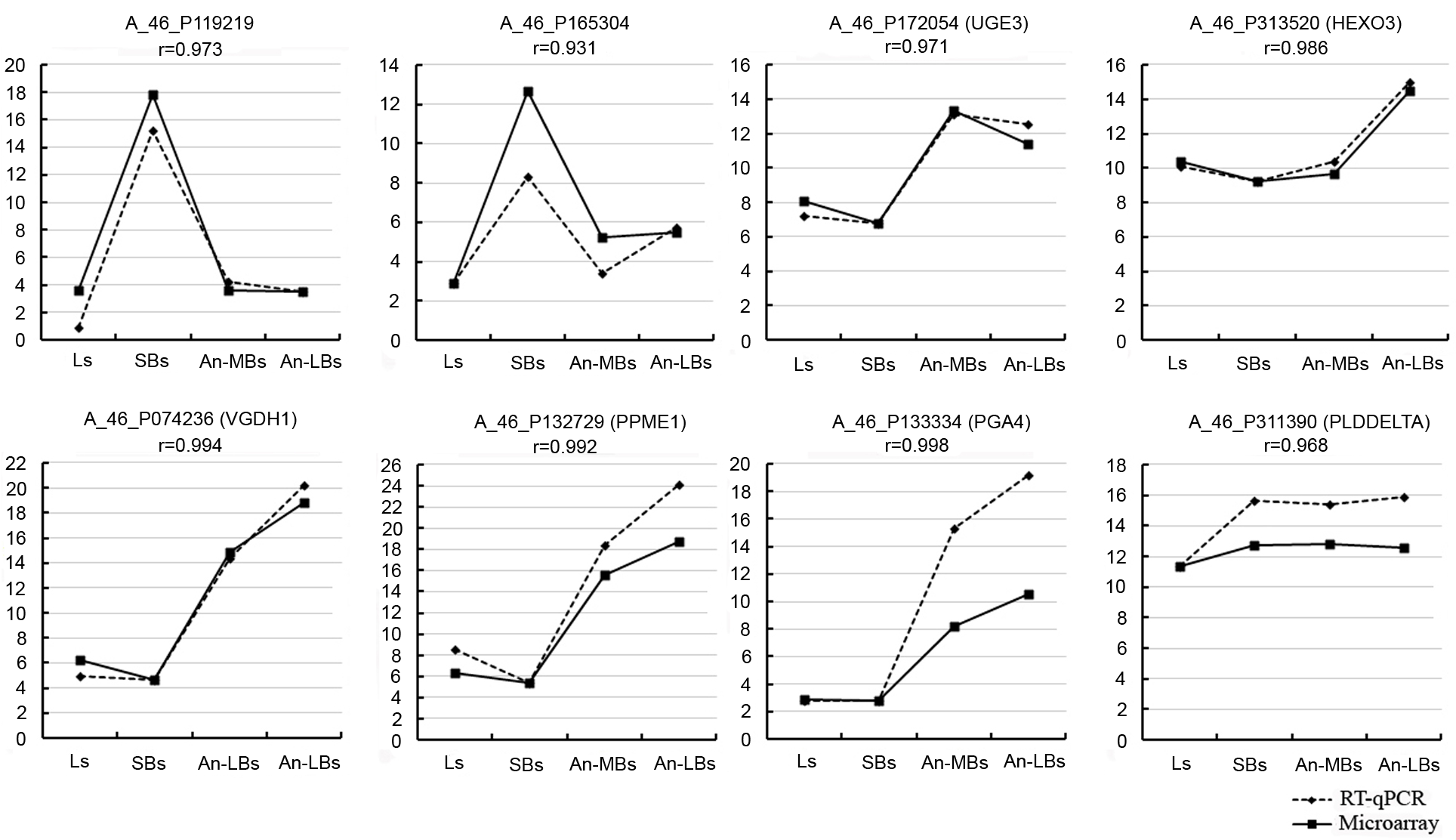

Supplement: S4 Fig — Three biological replicates were taken for both RT-qPCR and microarray analysis. The Y axis represents normalized log2 transformed expression values obtained using microarray analysis and RT-qPCR, respectively. The RT-qPCR data have been scaled such that the minimum expression value of RT-qPCR equals that of the minimum value of the microarray to ease profile matching. The correlation coefficient (r) between the two expression profiles is also indicated. Expression of B. napus β-actin (accession no. AF111812.1) was used as an internal control to normalize the RT-qPCR data. (TIF) [file pone.0154039.s004.tif]
